# A robust electrochemical immunosensor based on core–shell nanostructured silica-coated silver for cancer (carcinoembryonic-antigen-CEA) diagnosis

**DOI:** 10.1039/d0ra09015h

**Published:** 2021-03-09

**Authors:** Priyanka Singh, Pranav K. Katkar, Umakant M. Patil, Raghvendra A. Bohara

**Affiliations:** D. Y. Patil Education Society (Institution Deemed to be University) Kolhapur (M.S) India raghvendrabohara@gmail.com Raghvendra.Bohara@nuigalway.ie; CÚRAM, SFI Research Centre for Medical Devices, National University of Ireland Galway Ireland

## Abstract

This work addresses the fabrication of an efficient, novel, and economically viable immunosensing armamentarium that will detect the carcinoembryonic antigen (CEA) typically associated with solid tumors (sarcomas, carcinomas, and lymphomas) and is used as a clinical tumor marker for all these malignancies. We synthesized silver nanoparticles by single-step chemical reduction and coated with silica using a modified Stober method to fabricate silica-coated silver core–shell nanoparticles. The morphologies, structure, and size of the nanoparticles were characterized by Transmission Electron Microscopy (TEM), UV-Visible spectroscopy, X-ray diffraction (XRD), Raman spectroscopy, Fourier Transform Infra-Red Spectroscopy (FTIR), and Dynamic Light Scattering (DLS), respectively. The results indicated that the average size of Ag nanoparticles and silica-coated Ag nanoparticles is 50 nm and 80 nm, respectively. Our TEM results indicate that the silica-shell uniformly encapsulates silver core particles. Further, a disposable electrochemical immunosensor for carcinoembryonic antigen (CEA) was proposed based on the antigen immobilized in a silica-coated silver core–shell nanoparticle film on the surface of an indium–tin–oxide (ITO) flat substrate. The morphological characteristics of the constructed biosensor were observed by scanning electron microscopy (SEM) and electrochemical methods. Electrochemical impedance spectroscopy (EIS) and cyclic voltammetry (CV) were employed for the characterization of the proposed bioelectrode. The cyclic voltammogram appears to be more irreversible on silica coated silver core–shell nanoparticles. It is found that the fabricated immunosensor shows fast potentiometric response under the optimized conditions. The CEA could be determined in the linear range from 0.5 to 10 ng mL^−1^ with a detection limit of 0.01 ng mL^−1^ using the interface. The developed flat substrate of ITO for CEA detection (the model reagent) is a potentially promising immunosensing system, manifests good stability, and allows batch fabrication because of its economic feasibility.

## Abstract 2

The process flow of disposable electrochemical immunosensor fabrication.

## Graphical abstract

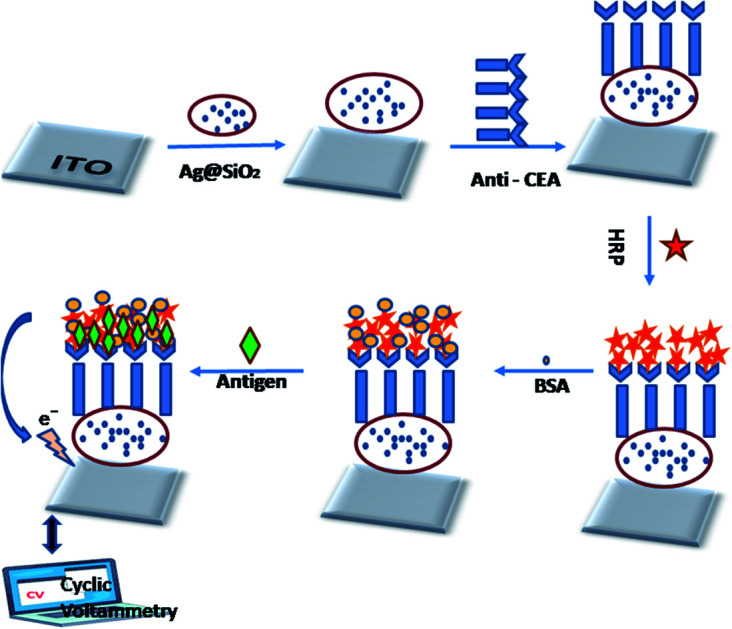

## 1. Introduction

Nanotechnology is the field of handling matter at the molecular and atomic scale and with developments in nanotechnology, there has been a remarkable rise in the use of nanoparticles in the biomedical field, especially in diagnosis. As per the research carried out by WHO, casualties from cancer in 2018 were 9.6 million, which is supposed to increase by 29.5 million by 2040. In 2018, out of all patients who died from cancer, 2.09 million cases were of lung cancer, 2.09 million cases were of breast cancer, and 1.80 million were of colorectal cancer.^1,2^ In all aforementioned cancer cases, tumor markers could have played a pivotal role in the early detection of these solid tumor cases.^3–5^ As reported to date, most human cancers (85%) are solid tumors. These include cancers of the colon, brain, ovary, breast, prostate, rectum, bladder, and other tissues. Even though there is a remarkable development in anti-cancer treatments, the effective treatment of cancers with solid tumors is not available.^6^ Hence, there is a possibility that if the tumor is diagnosed^7^ in the initial state,^8^ its can be treated well in advance.^9,10^ This might increase the time frame of life expectancy of the patient.^11–13^

Over the years, various immunoassays for the detection of tumor markers and better management of cancer have been reported, which include radiometry immunoassays, dynamic-light-scattering (DLS)immunoassays, colorimetric enzyme immunoassays,^14,15^ and other conventional immunoassays such as ELISA (enzyme-linked immunosorbent assay) and IHC (immunohistochemistry). The immobilization of antibodies on solid supports enables effective analyte capture in these assays as in the immunosensor. Nevertheless, as reported by Sakamoto *et al.*, these are cumbersome methods and there are the drawbacks of using these techniques as test results are not always accurate, require an exorbitant amount of reagents, have high possibility of damaged or false results, and are labor intensive.^16^ According to Wafik El-Deiry *et al.*, in 2019, excellent cancer care needs futuristic molecular diagnostics.^17^ Electrochemical immunoassay has gained approval and is being widely used to determine tumor markers due to its intrinsic advantages such as high sensitivity, selectivity,^18^ convenient label-free manipulation, miniaturized size,^19^ low cost, and fast analysis.^20,21^ There are various types of electrochemical immunosensors based on amperometry, electrochemical impedance spectroscopy, potentiometry, and conductometry.^22^ Developing a CEA immunosensor with good sensitivity and selectivity but without a complicated fabrication process still arouses researcher's considerable interest.^23^

A well-known method to monitor cancer is the screening of serum tumor markers.^24–26^ Nowadays, there are various tumor markers (*e.g.*, AFP, PSA, CA-125, HCG, CA-15-3, and CA19-9) available in routine clinical use for diagnosis.^27^ Among all the tumor markers, CEA is approved by the US Food and Drug Administration (FDA) and is an established tumor-associated antigen (TAA).^13,28^ According to Zhu and Basu *et al.*, CEA a glycoprotein with a molecular weight of about 180–200 kDa, comes from the CEA-related cell-adhesion (CEACAM) superfamily. It is commonly produced during fetal development but production stops before birth. CEA is expressed at deficient levels in various normal tissues including prostrate, tongue, cervix, oesophagus, tongue, colon, and stomach. High serum CEA level is related to carcinomas.^23,29^ CEA is expressed on the apical surface and the luminal segment of normal epithelial cells due to physiological conditions. In cancer tissues, CEA loses the polarized distribution, overexpresses itself, and is split from the surface of the cancer cells by phospholipase, which is evolved in the elevated level of serum CEA. Some other existing medical conditions can also raise CEA level, including inflammatory bowel disease, pancreatitis, infections, smoking, and cirrhosis of the liver.^28^ Pommerich *et al.* reported that CEA plays a crucial role in the adhesion, migration, and invasion of cancer and, based on its various extraordinary properties, it has become a significant target for immunotherapy and treatments based on antibody in CEA-positive solid tumors.^30^ In cancer patients, monitoring of the CEA level before surgery is also recommended by the American Society of Clinical Oncology (ASCO).^24,31^ As suggested by Bulut *et al.*, the level of CEA can also be elevated in emphysema, which is a lung condition, grouped under the more general term chronic obstructive pulmonary disease (COPD).^32,33^ It is essential to mention that people with COPD have a higher risk of more severe illness from COVID-19 due to their existing lung problems.^34–36^ Therefore, good workflow is needed for the proper diagnosis of patients with solid tumors.^8^

Recently, an indium–tin–oxide (ITO or InSnO_*x*_)-coated glass substrate has been reported, which is a miniaturized lab-on-a-chip related concept that is an n-type highly degenerated and wide energy gap semi-conductor, and is sturdy and metamorphic.^37^ The application of the ITO electrode has acceptability based on its user-friendly characteristics, which are its unique optical transparency, wide electrochemical working window, high electrical conductivity, excellent substrate adhesion, stable electrochemical and physical properties, conveniently etched, patterned, microarrayed, and its low cost.^38,39^ The use of ITO for sensing applications can be prominently enhanced by introducing nanoparticles on its surface.^40^ Metal nanoparticles provide a biocompatible microenvironment for biomolecules and significantly increase the surface-to-volume ratio of an immobilized biomolecule on the electrode surface,^41,42^ both of which ultimately influence electrical signal enhancement.^43^ Analysts in the field of nano-biosensors are always enthusiastic about finding new materials with suitable properties to enhance the behavior of biosensors.^44^ Composite nanoparticles are suitable for forming a continuous electric field and for increasing the transferred ratio of the electrons compared to single nanoparticles. Thus, they can effectively fasten the regeneration process of sensors.^45,46^

The present paper aims to merge the advantages of metal nanoparticles, *i.e.*, silver, with silicon dioxide (SiO_2_) on an ITO flat substrate to design a novel electrochemical immunosensor for the sensitive and selective detection of CEA. Ag nanoparticles were prepared by an improvised chemical reduction method in the present work using citrate and then the coating of Ag nanoparticles was carried out with silica by the modified Stober method (sol–gel).^40,47,48^ According to the work done by Thorat *et al.*,^49^ silica provides a biocompatible environment. The electrochemical experiments were carried out by cyclic voltammetric (CV) studies. In addition, electrochemical impedance spectroscopy (EIS) was also a part of the study and has been implied to observe the electron transfer resistance. EIS is a faradic impedance method, which is carried out in the presence of a redox couple. It is a label-free technique to determine the antigen–antibody interactions on the electrode surfaces by measuring their capacitance and interfacial charge transfer resistance. As soon as a target protein binds to the pre-functionalized probe surface, the impedance of the electrode–solution interface changes, which are detected electrically over a range of measurement frequencies.^38,50^

## 2. Experimental

### 2.1 Materials and methods

All the following chemicals were purchased from Sigma-Aldrich and used without further purification: silver nitrate (AgNO_3_), trisodium citrate dehydrate (Na_3_C_6_H_5_O_7_·2H_2_O), tetra-ethyl-*ortho*-silicate (TEOS), absolute ethanol, ammonia solution (28–30 wt%), bovine-serum-albumin (BSA, 99%), HCl, NaOH, HRP (horse-radish-peroxidase), CEA antigen and the corresponding antibody, ITO-glass-slides 2 × 2 cm^2^ (resistance 30 Ω), and acetone. The buffer for the assay was 0.1 M phosphate-buffered saline (PBS), which was prepared by mixing the stock standard solutions of dibasic sodium phosphate (Na_2_HPO_4_) and monobasic sodium phosphate (NaH_2_PO_4_). All aqueous solutions were prepared with deionized double distilled water (18.2 MΩ). All the glass apparatus were properly cleaned with detergent and then washed with deionized water before being used for the experiment.

### 2.2 Equipment

An EVO-18 CARL ZEISS special edition instrument obtained the scanning electron microscopy (SEM) images. The DLS measurements of both the sample were performed using an NICOMPTM 380 ZIS (Santa Barbara, CA, USA) to determine the hydrodynamic diameter (HDD). Cyclic voltammetry and electrochemical impedance measurements were performed on a ZIVE MP1 electrochemical workstation. Transmission electron microscopy (TEM) was carried out to study the morphology and the size of the Ag NPs and Ag@SiO_2_ NPs. For this purpose, we used Philips CM 200 model TEM and operating voltage in the range of 20–200 kV with 2.4 Å resolution. All Fourier-transform infrared (FTIR) spectroscopic measurements were performed on an Alpha ATR Bruker Spectrometer (KBr pressed disks). The UV-Vis absorbance and fluorescence measurements were recorded on a Shimadzu (Model no.·UV 1800) double beam spectrophotometer in the wavelength range of 200–1100 nm. XRD measurements were carried out using a Rigaku Miniflex 600 X-ray diffractometer with Cu Kα radiation at room temperature operated at a voltage of 30 kV and a filament current of 40 mA in the range from 20° to 80°. To measure the size of the nanoparticles accurately, each peak was Gaussian fitted and the instrumental broadening was subtracted using Si standard sample broadening. The crystallite size of both the nanoparticles was calculated from the FWHM of the highly intense diffraction peak using the Debye Scherrer formula*D* = 0.9*λ*/*β* cos *θ*where, *D* is the average crystallite domain size perpendicular to the reflecting planes, *λ* is the X-ray wavelength, *β* is the FWHM of the diffraction peak, and *θ* is the diffraction angle.^51^ The Raman spectra were obtained using an inVia Renishaw micro Raman spectrophotometer. The system was automatically calibrated against silicon wafer peak at 560 cm^−1^. Raman scattering was excited by a continuous wave argon laser with a power of 100 MW. The spectra were measured at temperatures of 300 and 78 K.

### 2.3 Synthesis of Ag NPs

Silver colloids were prepared according to the chemical reduction method by the modified version of the well-known Turkevich method.^52^ Briefly, 1.5 mM of AgNO_3_ was added to 98 mL distilled water and appropriately boiled under vigorous stirring. Then, 5 mL of freshly prepared 1% TSC (trisodium citrate) was added within 2 min into the AgNO_3_ solution and then, the mixture was stirred at 100 °C continuously. Later, after blending for 1 h, the mixture was kept to chill at RT (room temperature) for another 10–15 h for proper seeding. The purification of the colloidal solution was carried out by centrifuging the suspension at 10 000 rpm for 20 min. After collecting the silver pellet, it was washed twice and some distilled water was later added, followed by storage at RT for further experiments.

### 2.4 Synthesis of Ag@SiO_2_ NPs

The silica coating on Ag NPs were done by modified “Stober” method.^47^ The as formed concentrated silver nanoparticles were put into mixture of pure ethanol (100 mL, 99.9%), liquid ammonia (28 to 30%), and deionized water. After 35 min of sonication, various amount TEOS (tetraethyl orthosilicate) were added dropwise (gradually starting from 2 to 15 μL) then the mixture was correctly agitated for 15 h at ambient RT. By altering the amount of the TEOS, the diameter of silica coating can be managed. To segregate the mixture, centrifuged it at 13 500 rpm for 40 min followed by washing thrice in ethanol (100%) for 15 min at 8000 rpm. For use in future purposes, the obtained Ag@SiO_2_ nanoparticles were re-dispersed in purified water.

### 2.5 Framing of the electrochemical immunosensor

The graphic representation of the manufacturing method of the immunosensor is illustrated in Fig. 6. The sonication of the ITO glass slides (2 × 2 cm^2^) was carried out in acetone, ethanol, as well as water sequentially for about 30 min, then dried beneath a flow of nitrogen. 10 μL Ag@SiO_2_ nanoparticles were plunged on the facet of the pre-cleaned ITO and then left to wither at RT overnight. Further, 10 μL of 10 μg mL^−1^ anti CEA solution was dropped at the Ag@SiO_2_ NPs/ITO surface for 8 h at 25 °C. After that, 15 μL 6 μg mL^−1^ HRP was utilized to obstruct the non-specific sites on the antibody-altered facet of the electrode. There are other advantages of using the HRP enzyme as it can magnify a weak sign and elevate the degree of detection of a selected biomolecule. BSA (1%) was added to the electrode surface as a blocker of the excess active group. After the drying of the electrode surface, varying concentrations of the CEA solution were casted on the facet of the electrode. Further, the altered ITO (electrode) was kept for incubation at 4 °C for 1 h and later cleaned by the use of the prepared buffer solution in order to remove the excess CEA. It is essential to mention here that the electrode was dried by nitrogen following each fabrication step.

### 2.6 Electrochemical detection

Ambient laboratory temperature, *i.e.*, 25 °C was chosen for all the electrochemical tests. Besides, pH 6.0 phosphate buffer solution (PBS) was utilized as the electrolyte. Electrochemical tests such as CV and EIS studies of the bare ITO and the altered ITOs was executed using 0.1 M pH 6.0 PBS buffer consisting of Na_2_HPO_4_ and NaH_2_PO_4_ (the pH was maintained by HCl and NaOH).

### 2.7 Electrochemical experimentation

All the experiments related to electrochemical quantification were accomplished utilizing a ZIVE MP1 electrochemical workstation, which has a standard three-electrode set up consisting of altered ITO as the functioning electrode, platinum cable as the counter electrode, and saturated-calomel electrode (SCE) as the reference electrode.

## 3. Results and discussion

### 3.1 Characterization of Ag nanoparticles and Ag@SiO_2_ nanoparticles

**Fig. 1:**
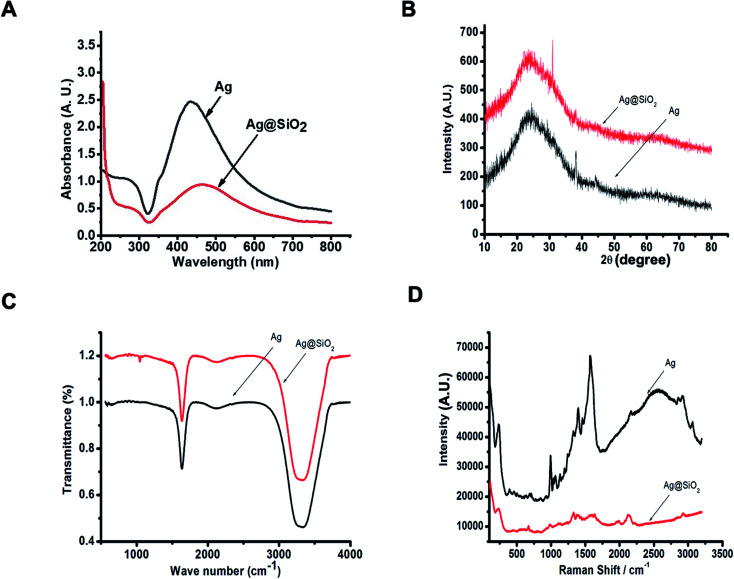
(A) UV-visible spectrum of Ag NPs and Ag@SiO_2_ NPs. AgNPs exhibited a sharp characteristic peak at 420 nm in the UV-visible absorption spectra and after the deposition of the silica layer on the Ag nanoparticles, the peak shifted to longer wavelength. (B) XRD pattern of Ag NPs and Ag@SiO_2_ hybrid nanoparticles. (C) FTIR spectra of Ag nanoparticles and Ag@SiO_2_ nanoparticles show that the peaks become wider after the coating of silica. (D) The Raman spectra of Ag nanoparticles and Ag@SiO_2_ nanoparticles demonstrate the expansion of the peak after coating.

The prepared silver nanoparticles were obtained by employing trisodium citrate dihydrate (Na_3_C_6_H_5_O_7_·2H_2_O) as a capping as well as a reducing factor.^52^ The layering of silica on the outer surface of silver was implemented by a modified “Stober” (sol–gel) procedure.^47^ Our first observation was that the stability of the silica shells depended on the conditions under which they were formed. The initial change in the color from yellow to black indicates the evolution of steady Ag nanoparticles. Ammonia (28–30 wt%) was used as the catalyst of the reaction and it was essential to acquire a proper coating. Low ammonia concentration can lower the reaction rate to some scale. It was discovered that ammonia and water concentration command the balance between the hydrolysis of TEOS and the condensation of its hydrolyzed monomers.^53^ The structure and properties of Ag nanoparticles and silica-coated Ag nanoparticles were distinguished by UV-Vis spectroscopy, FTIR, XRD, and TEM. It is shown in Fig. 1(a) that after the deposition of the silica layer on Ag nanoparticles, the peak shifted to longer wavelengths. The shell thickness after coating with silica in terms of the SPR (surface plasmon resonance) signal was attributed to the growing local refractive index of the nanoparticles and due to this, red shifting of the peaks took place.^54^ XRD diffraction was later used to investigate the difference in the crystal structure between the two nanoparticles.^55^ For the XRD pattern of Ag nanoparticles and Ag@SiO_2_ nanoparticles, both the samples were plunged on the facet of the glass and dried in air, and then the measurements were carried out. The phase identification of the samples reported is accomplished by the corresponding peak locations as well as matching the intensities in the XRD patterns to the patterns given in the JCPDS (Joint Committee on Powder Diffraction Standards) data collection. The XRD pattern of the Ag nanoparticles is depicted in Fig. 1(b), which was recorded in the range from 10° to 80°. There are three characteristic diffraction peaks at 38.2°, 44.03°, and 64.2°, which are analogous to the (111), (200), and (220) Bragg reflections of the face-centered-cubic (fcc) crystal shape of silver nanoparticles, respectively. Few other peaks are also visible because of the presence of carbon, which was related to the biomaterial. The peaks for the (200) and (220) planes are slightly intense, whereas the peak for the (111) plane is more intense. Fig. 1(b) also shows the XRD pattern of the silica-coated silver nanoparticles. The weak and extensive peak at the 2*θ* of about 22° can be assigned to a definite layer of silica.

To determine the functional groups, we subjected the colloidal solution to FTIR analysis. The sole purpose of FTIR was to study the formation of the silica layer on the Ag nanoparticles. When we observe the FTIR of Ag nanoparticles, there are visible peaks at 1637.73 cm^−1^, 2119.06 cm^−1^, and 3331.39 cm^−1^. Also, in the FTIR spectrum of Ag@SiO_2_ nanoparticles, the data revealed that the peaks attributed to 1638.48 cm^−1^ and 2123.94 cm^−1^ may be ascribed to the presence of silica on the surface of the particles (Si–O–Si and Si–O). In addition, the band at 3340.79 cm^−1^ was ascribed to C–N stretching, which represents the aliphatic amine. Further Raman spectroscopic analysis was implemented in order to learn the vibrational characteristics of the core–shell particles. Nowadays, this spectroscopic analysis is widely used in cancer diagnosis. As we know, colloidal nanoparticles are susceptible to chemical instability and Raman spectroscopy is significantly less susceptible to water interferences.^56^ It was found that the synthesized particles exhibited good stability. Ag nanoparticles show two large peaks at approximately 1569 cm^−1^ and 1396 cm^−1^, whereas Ag@SiO_2_ nanoparticles show much smaller scattering at 1630 cm^−1^ and 1328 cm^−1^.

**Fig. 2:**
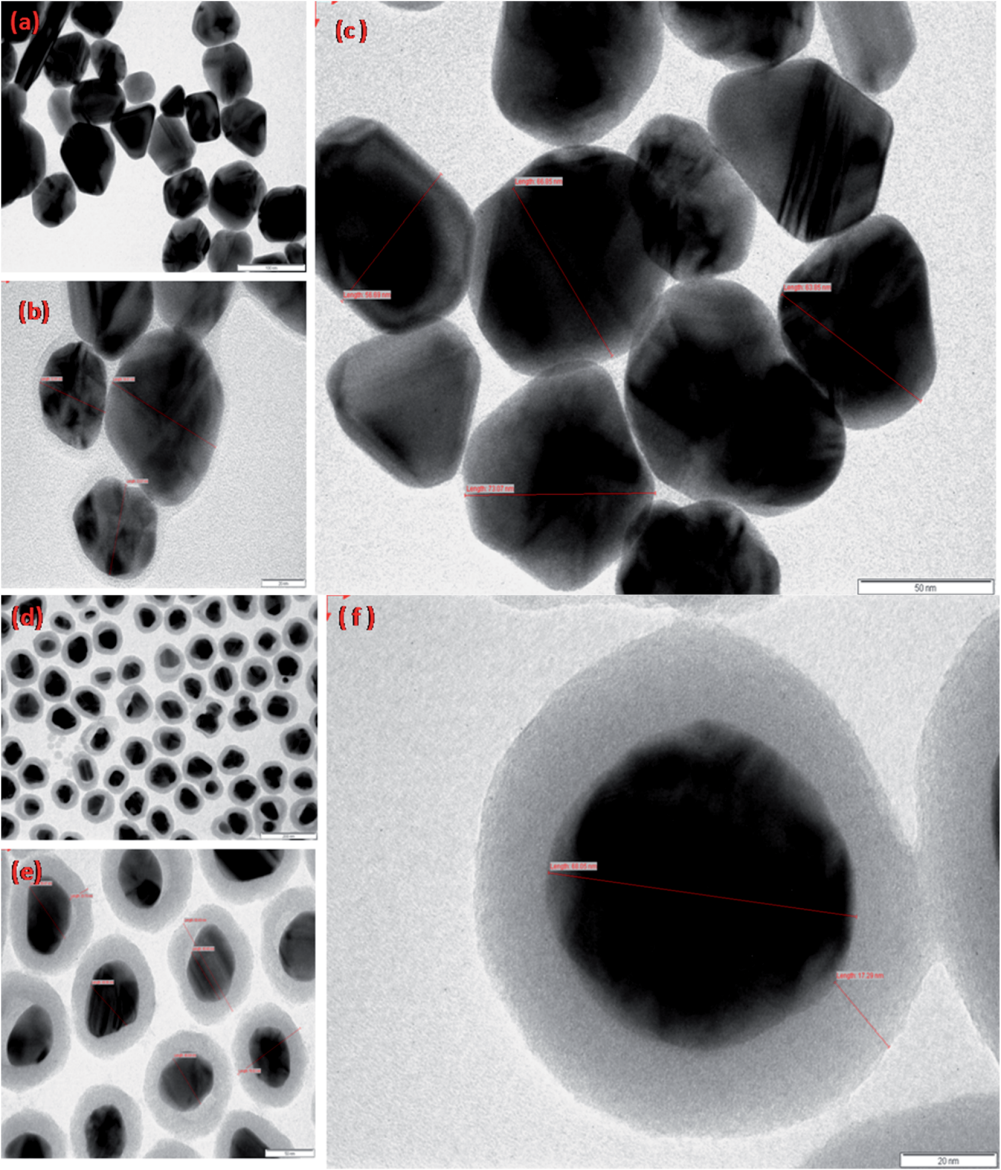
TEM images of the prepared (a)–(c) Ag nanoparticles and (d)–(f) Ag@SiO_2_ nanoparticles shown in the TEM images at different magnifications. As one can see, the Ag nanoparticles have a spherical shape with the average size of 50 nm, while the TEM images of Ag@SiO_2_ nanoparticles show uniform, clean, and distinct coating of silica on the surface of the Ag nanoparticles; the Ag@SiO_2_ nanoparticles have an average size of 80 nm.

TEM analysis was carried out to examine the configuration and volume of the Ag nanoparticles and the Ag@SiO_2_ nanoparticles. Fig. 2 exhibits the TEM images of Ag nanoparticles and silica-coated Ag nanoparticles at various magnifications. To accomplish this, both the nanoparticles were separately dropped on to a carbon-coated copper grid and dried in air, followed by scanning the grid employing a Philips CM 200 model transmission electron microscope at the operating voltage of 20 to 200 kV with a resolution of 2.4 Å. Extensive size distribution was observed for the silver nanoparticles, starting from 30 to 100 nm with a mean size of 50 nm, while the average size of the Ag@SiO_2_ nanoparticles was 80 nm.

**Fig. 3:**
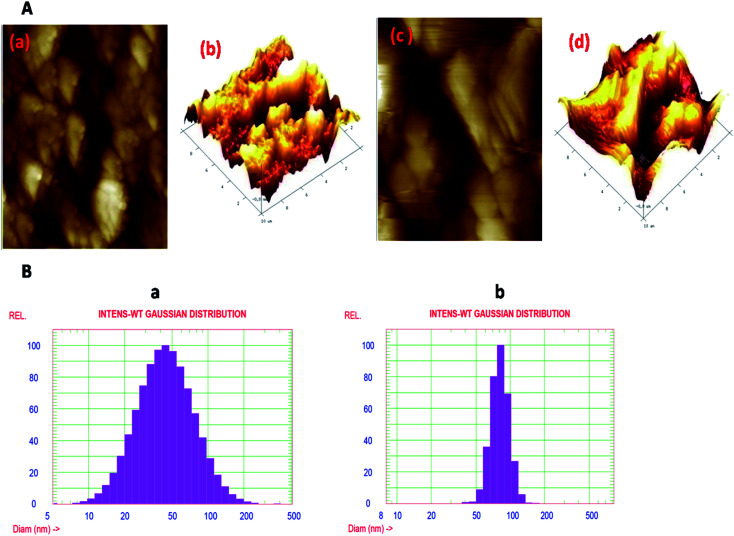
(A) The topological properties of Ag nanoparticles and Ag@SiO_2_ nanoparticles, which were examined by AFM. (B) The hydrodynamic-broadness of both the nanoparticles, which was decided by the use of DLS histograms. (a) The DLS of Ag nanoparticles and (b) the DLS of the Ag@SiO_2_ nanoparticle colloidal solution. The stability of both the nanoparticles was measured by zeta-potential measurements (not shown here), employing a zetasizer. The zeta-potential of the Ag nanoparticles and Ag@SiO_2_ nanoparticles was −27.7 mV and −21.7 mV, respectively.

Topological studies of both the nanoparticles were carried out by AFM on glass slides. The images are shown in Fig. 3. The images are assembled in a torsion manner and the interface can be seen. The AFM images illustrate the matching height description of the prepared silver and Ag@SiO_2_ nanoparticles. The maximum number of Ag nanoparticles are detected in the size span from 30 to 80 nm. Several larger particles are also present according to the study of the AFM data but, according to histogram scanning, their number is limited. The height profile of the nanoparticles, as suggested by line analysis, is in the same range, as confirmed by TEM analysis. From the AFM images, it is visible that once coated with silica, the size of the nanoparticles increased, *i.e.*, approx. 80 nm. Both the nanoparticles' stability was measured in terms of the zeta potential using a zetasizer. The prepared colloidal nanoparticles maintained a net negative charge on their facet and stayed in solution due to joint electrostatic-repulsive force. It is understood that because of the adherent citrate ions, Ag nanoparticles have a negative charge; simultaneously, a repulsive potential drive the particles in order to prevent aggregation. Hence, there is no need for any additional stabilizing agent and the particles in the suspension remain steady. It was found that the incorporation of even a tiny amount of silica promptly reduces the zeta potential. The colloidal stability of the Ag nanoparticles is −27.7 mV and that of the Ag@SiO_2_ nanoparticles is −21.7 mV. This means that the colloidal stability of Ag nanoparticles decreases after coating. Generally, higher the zeta potential, the more stable the colloid.

**Fig. 4:**
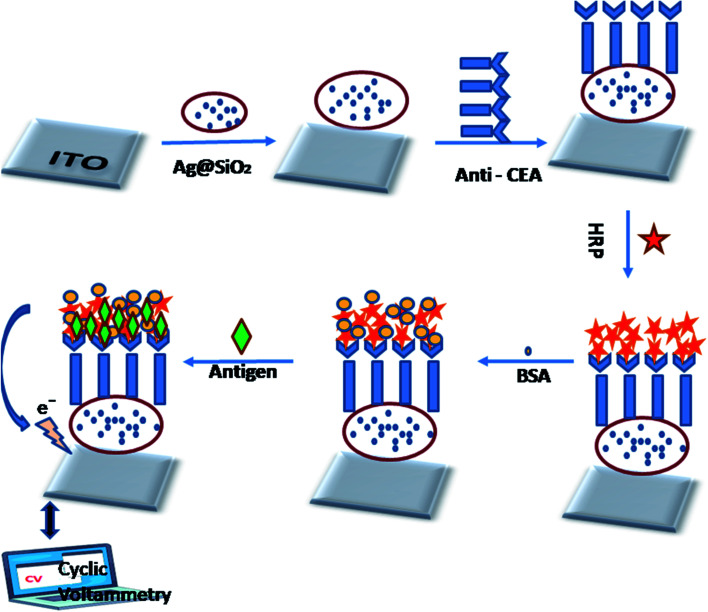
Process flow for disposable electrochemical immunosensor fabrication.

The hydrodynamic diameter of the Ag nanoparticles was determined using DLS, which tells us about the size distribution of the nanoparticles. Fig. 3(B)-(a) represents the DLS of the Ag nanoparticle colloidal solution, which shows the formation of adequately distributed nanoparticles in the range from 10 to 200 nm, besides a mean volume of 50 nm. On the other hand, Fig. 3(B)-(b) shows the DLS of the Ag@SiO_2_ nanoparticle colloidal solution, demonstrating the increase in the size of the nanoparticles from 50 to 200 nm, besides a mean diameter of 80 nm. The DLS results are almost similar to the TEM results (Fig. 4).

**Fig. 5:**
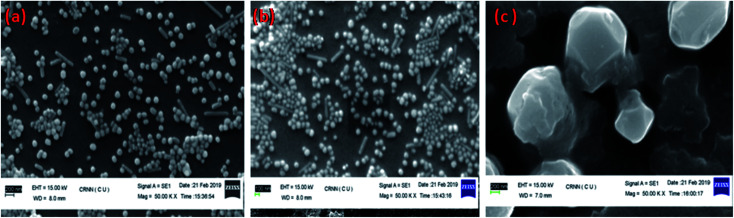
SEM of (a) Ag@SiO_2_ nanoparticles on ITO film, (b) CEA/HRP/anti-CEA/Ag@SiO_2_ NPs at Mag 1.00 hX, (c) CEA/HRP/anti-CEA/Ag@SiO_2_ NPs at Mag 5.00 kX.

The effectiveness of an electrochemical immunosensor is intertwined to its physical framework.^57,58^ Consequently, the arrangement of the Ag@SiO_2_ nanoparticles film is an essential component. SEM perceived the configuration of the facet of the film and in Fig. 5, it is observed that the Ag@SiO_2_ nanoparticle film exhibited a spotless and identical web arrangement. However, the SEM image of the anti-CEA/HRP/CEA/Ag@SiO_2_ NPs/ITO film exhibits the captured biological molecule's profusion by systematic allotment. Consequently, in the immobilization of the anti-CEA, the presence of the Ag@SiO_2_ nanoparticle film has a major role with the HRP enzyme and ultimately for detection of the CEA antigen.

**Fig. 6:**
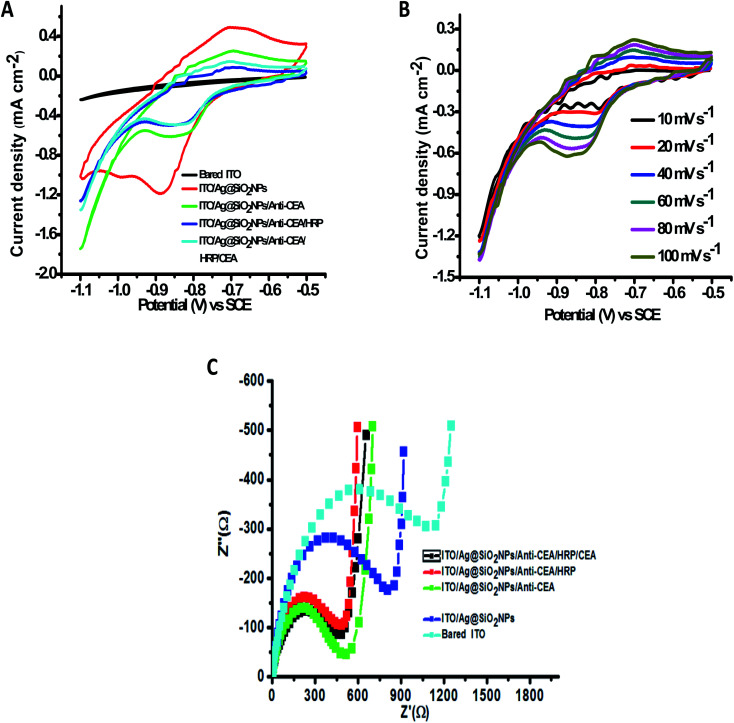
(A) CV of the stepwise immunosensor fabrication process at a scan rate of 60 mV s^−1^ using 0.1 M pH 6.0 PBS buffer solution, which contains Na_2_HPO_4_ and NaH_2_PO_4_. (B) CV of the fabricated immunosensor at different scan rates. (C) EIS related to the various steps elaborated in the manufacturing process.

Immobilized antibody methodology is the foremost method for developing effective diagnosis and separation tools.^40,59^ The methods of immobilizing biomolecules have been modified, and we look at the biochemical and biological matrices. However, capturing an analyte of interest in these compound patterns with a high degree of sensitivity and specificity has significant importance. Because of this very fact, the antibody is a class of biorecognition molecules that are exactly tied up with their corresponding antigen, which is highly required in the advancement of immunodiagnostic methods. There are other parameters and specificity, which are the foremost trait of a perfect immunoassay system, for instance, high sensitivity and lower limits of detection. Two main methods to study the Janus-faced properties of the altered electrode are electrochemical impedance spectroscopy and cyclic voltammetry.^26^ The current response was significantly elevated compared with that of bare ITO when Ag@SiO_2_ nanoparticles were immobilized on it subsequently, indicating that Ag@SiO_2_ nanoparticles possess excellent electrochemical characteristics. The current response decreased after the payload of CEA antibody, HRP, and CEA was sequentially loaded onto the altered electrode facet effectively, which could be ascribed to the stoppage of the bio-macromolecules. It was observed that there is a reduction peak at −1.7 mA and an oxidation peak at 7.5 mA at a scan-rate of 60 mV s^−1^. There is a particular binding of CEA to its corresponding antibody, which leads to the obstruction of electron transfer; when there is an increase in the CEA concentrations, the peak-current intensities increase. Fig. 6 shows the CV of the electrochemical immunosensor when we used PBS buffer, keeping the pH at 6.0. A couple of peaks appear after the binding of Ag@SiO_2_ nanoparticles on the ITO flat substrate, which can be ascribed to the redox reaction. The reduction in the peak-current responses takes the place at both the cathode and the anode, following the immobilization process of the CEA antibody, which indicates that the steric hindrance effect occurred and weakened the electron transfer between the electrode and the solution. When HRP binds, it can be seen that the anodic peak current decreases and cathodic peak current reaction increases because HRP amplifies even a weak signal and elevates the selected molecule's level of detection. This is a typical electro-catalytic process. Once the CEA antigens were cast onto the electrode facet, they bound with CEA antibodies and the formed antibody–antigen composites could later result in the obstruction of the electron-transfer rate because of the sturdy steric hindrance effect, which leads to a decrease in the response of the peak current. The further stepwise manufacturing method of the immunosensor was also identified by electrochemical impedance spectroscopy, which validated that the facet of the electrode was refashioned favorably.

The EIS spectrum shows a straight line at low frequencies and a semi-circle curve at high frequencies. The *R*_et_ (electron transfer resistance) of the altered film was shown as per the semi-circle broadness. The bigger the semi-circle broadness (diameter), the larger the *R*_et_ rate. A variation in each step was observed on changing the semi-circle diameter. As one can see, the non-modified ITO solid flat substrate manifests a smaller semi-circle diameter (Fig. 6(C)), whereas Ag@SiO_2_ nanoparticles-altered electrode facet showed significantly larger peak currents and miniscule electron transfer when compare to the bare ITO electrode, and a very tiny semi-circle diameter of the Nyquist plot and several reversible redox peaks could be observed. The redox probe's ability to acquire the facet of the electrode was highly affected by the presence of Ag@SiO_2_ nanoparticles, which decreased the redox probe. The consecutive capture of anti-CEA, HRP, BSA, and CEA leads to a progressive growth of the *R*_et_ due to the insulation qualities that the protein possesses. It defines that the electrical conductivity of the electrode's altered facet was enriched because of the Ag@SiO_2_ nanoparticles, which have a large particular surface zone as the fine conducting element. After the addition of anti-CEA, an increase in the *R*_et_ and diminished peak currents were observed, which indicates the successful immobilization of the anti-CEA on AgSiO_2_ nanoparticles because of the EDC/NHS bonding as well as the pi–pi stacking interconnection between the nanoparticles and the proteins. The results indicated that CEA, HRP, anti-CEA, and the Ag@SiO_2_ nanoparticle film were conveniently altered on the surface of the ITO solid flat substrate in flow. These proteins have the capacity to block the electron transfer of the electrochemical probe efficiently. The parameters mentioned here can cause a large difference after the introduction of HRP, demonstrating that HRP could cover the non-specific sites on the altered electrode. Later, it was observed that there are diminished peak currents and an intensified Nyquist diagram post the capture of CEA-antigens. This suggests that the electron transfer rate between the electrode and the solution was greatly hindered because of the immuno-reaction of the antigen with its corresponding antibody. Both the mentioned outcomes were in agreement with the assumption that the electrode was altered as anticipated.

### 3.2 Optimization of the experimental conditions

**Fig. 7:**
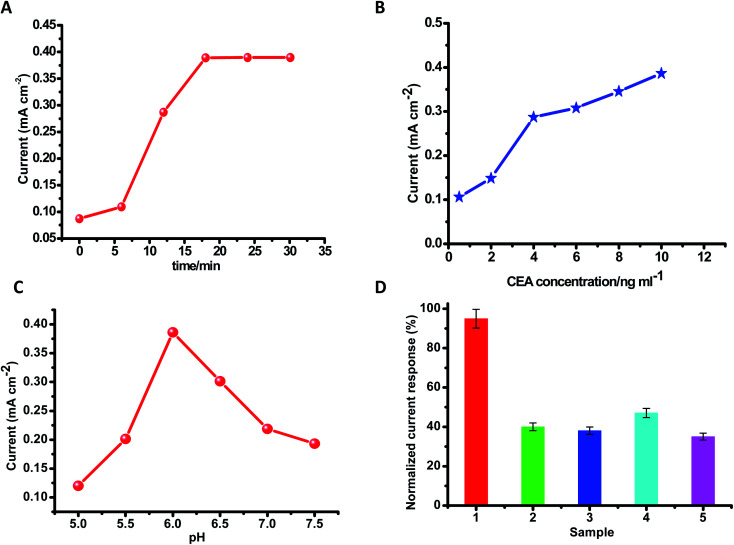
Graphs for optimizing the experimental parameters. (A) The effect of incubation time on the response signal at various time intervals. (B) Calibration plot of variation in the reduction current reaction of the fabricated immunosensor *versus* CEA. (C) Effect on the response of the fabricated immunosensor after varying the pH of the PBS buffer solution. (D) Selectivity (impact of obstruction by other factors) of the fabricated disposable immunosensor in the presence of (1) CEA (5 ng mL^−1^), (2) 5 ng mL^−1^ CEA + 20 ng mL^−1^ PSA, (3) 5 ng mL^−1^ CEA + 20 ng mL^−1^ AFP, (4) 5 ng mL^−1^ CEA + 20 ng mL^−1^ glucose, (5) 5 ng mL^−1^ CEA + 20 ng mL^−1^ CA-125.

There are several experimental specifications that need to be optimized in order to manufacture an immunosensor. The antigen (CEA) was optimized first to enrich the sensitivity. It was unveiled that even at low concentrations of CEA, well defined voltammetric peaks are obtained. Furthermore, with the increase in the concentration of CEA, the peak-current intensities increase. On the other hand, a significant enhancement in the concentration of CEA leads to a high signal and also follows the towering background at a similar time because of the non-specific adsorption process. It was monitored that the scale of the current *versus* CEA (Fig. 7(B)) is straight in the range from 0.5 ng mL^−1^ to 10 ng mL^−1^, and it is appropriate for the computable task. The highest signal-to-noise ratio was attained with 10 μL CEA. Thus, 10 μL of the antigen was chosen as the optimized condition. The limit of detection of this procedure was considered to be 0.01 ng mL^−1^. A string of 6 repeated quantifications in addition to the CEA concentrations in the range from 0.5 ng mL^−1^ to 10 ng mL^−1^ were taken.

Protein denaturation can occur if the pH is unsuitable. The electrochemical reactions in an immunosensor greatly influence the pH of the detection suspension. The pH of the solvent-buffer was explored thoroughly in order to obtain an excellent electric current signal. Maintaining the CEA concentration persistently (10 μL), the outcome of the pH value of the buffer solution on the intensity of the current was calculated by maintaining the pH in the range from 5.0 to 6.0 and after reaching the maximum current at pH 6.0, the intensity of the current reduced correspondingly once the pH-value increased from 6.0 to 7.5. This suggests that pH 6.0 is more advantageous to the sensor assembly system's current response. Thus, pH 6.0 was selected as the most favorable value. By using PBS-buffer at pH 6.0, aprecisely elaborated cyclic-voltammograms and fine response to the CEA were obtained. By maintaining the concentration of CEA at 20 ng mL^−1^, the impact of time of incubation was examined in the time span of 0–30 min. It was observed that within the first 17 min, Δ*I* quickly increased and once the incubation time was longer than 17 min, it came to a constant value. Thus, 17 min was identified as an excellent condition for incubation. Thinking about the practicability in the genuine way of life, room temperature was selected to carry out all the experiments.

### 3.3 Selectivity

The effect of interfering substances on the response of the CEA immunosensor was investigated. It was found that four interfering substances do not influence the current response to CEA, such as PSA, AFP, glucose, and cancer antigen 125. The current value was examined by combining 5 ng mL^−1^ CEA in addition to 20 ng mL^−1^ prostate specific antigen (PSA), 20 ng mL^−1^ alpha-fetoprotein (AFP), 20 ng mL^−1^ glucose, as well as 20 ng mL^−1^ cancer-antigen 125. There was no apparent change in the currents, if it is correlated with 5 ng mL^−1^ CEA, which shows outstanding specificity and selectivity of the disposable electrochemical immunosensing system for CEA (Table 1).

**Table 1:** Showing different materials along with the detection range and detection limit

Materials	Detection range (ng mL^−1^)	Detection limit (ng mL^−1^)	Ref.
Ba–NH–Au NPs (organoclay–nanogold composite) film	0.05–5.0; 5.0–120	0.01	60
CS-MWNT–Au NP composite	0.3–120	0.1	31
Carboxylated g-C_3_N_4_/TiO_2_ nanosheets	0.01–10.0	0.0021	61
HRP–anti-CEA–NGGN	0.05–350	0.01	62
Ng/chit/nano/Au composite	0.2–120.0	0.06	63
Thi@NPG/AuNPs	0.01–100	0.003	64
AuNP@Nafion/FC@CHIT	0.01–150	0.0031	65
NG/P-PB/nano-Au composite film	0.5–10 and 10–120	0.2	66
[Ag–Ag_2_O]/SiO_2_ nanocomposite material	0.5–160	0.14	67
CS–CNTs–GNPs nanocomposite film	0.1–2.0	0.04	68
Ag@SiO_2_ NPs	0.5–10	0.01	This work

### 3.4 Reproducibility and stability of the immunosensor

There are two prominent components in the evolution and implementation of the immunosensors, *i.e.*, reproducibility and stability. 5 ng mL^−1^ CEA along with 5 immunosensors were constructed identically to evaluate the reproducibility of the proposed immunosensor for the detection of the antigen. Also, we obtained a relative standard deviation (RSD) of 3.2%, which depicted that the immunosensor had good reproducibility. The stability of the immunosensor is a fundamental need for stable and desired analysis. The fabricated immunosensor was kept for six weeks at 4 °C and it was found that only a minute decrease in the current response occurred. Eventually, the current-response was examined after 2 weeks, 4 weeks, and finally, after 6 weeks. It was observed that the immunosensor retained 97.1, 96.4, and 94.8% of the earlier response, respectively. It shows that the developed immunosensor possessed good stability (Table 2).

**Table 2:** Showing different types of sensors and a quick comparison

Materials	Type of sensor	Key properties	Our key properties	Advantages	Ref.
Ag@SiO_2_–RuBpy	MEF based FRET aptamer sensor	Fluorescence sensing approach by using optimal fluorescence-enhancement	Based on impedance and cyclic voltammetry results	More accurate and less time consuming	69
ZrO_2_–Ag–G–SiO_2_ and In_2_O_3_–G–SiO_2_	Mesoporous electrochemical immunosensor	FTO (fluorine doped tin-oxide) based sensor	We used ITO (indium tin oxide) for sensing	ITO provides smoother surface and higher transparency at a given conductivity than FTO	70
Ag@SiO_2_	Sandwich type immunosensor for the detection of *E. coli* 0157:H7	DPV (differential pulse voltammetry) based sensing. Stripping current response of the sensor was 60 min	Stripping current response reached a constant value only after 17 min	Requires less incubation time and as a result, provides fast analysis	47
Ag@m-SiO_2_	SERS signal based sensor	Demonstrates SERS sensing towards pesticides	Excellent voltammetric benefits, straightforward method employed	More elaborate study of the sensing system	71
Au@Ag@SiO_2_–Au NP	SERS based immunoassay of AFP	Sandwich immunoassay strategy by using the nitrocellulose membrane	There is a visible impedance spectrum (*R*_et_) and cyclic-voltammograms are shown after each fabrication step	We used an extremely simple and less intricate process	72
CdTe quantum dots decorated Ag@SiO_2_ NP	SHINEFs (shell isolated NP-enhanced fluorescence) for the detection of tetracycline	Fluorescence based detection of tetracycline with the SHINEF effect	We kept the sensing system simple using only silica and silver	Simple and cost effective	73
Fluorescent dual labelled Ag@SiO_2_ NP	Optical immunosensor	GPTMS coated quartz glass based optical immunosensor	We used ITO flat substrate	Eliminates the demand for an over-coating of the conductive layer	74
Multishell Au@Ag@SiO_2_ nanorods	Electrochemical sensor	DPV based theobromine (THB) quantification. Glassy carbon electrode used (GCE restricts the use of measurements at on-site)	We utilized ITO as a disposable electrode substrate	It can be reproduced in batches	75
Polystyrene sphere@Ag/SiO_2_/Ag	Detection of HCC (hepatocellular–carcinoma) biomarker	Used DSNB (5,5-dithiobis succinimidyl-2-nitrobenzoate) molecule as the linker for detection of HCC	We adopted the HRP enzyme, which is easily available	Cost effective and pliable sensing system	76

## 4. Conclusion

The novel electrochemical disposal immunosensor was fabricated by immobilizing anti-CEA on Ag@SiO_2_ nanoparticle-coated ITO solid flat substrate to detect CEA. We observed that due to the presence of silica-coated silver NPs on the surface of the electrode, there are excellent voltammetric benefits, good stability, and exceptional biocompatibility towards the detection, which, at the same time, provides comprehensive binding sites for the immune reaction. A dynamic connection between the stripping current response of the modified ITO surface and the logarithmic rate of CEA concentration was found to range from 0.5 ng mL^−1^ to 10 ng mL^−1^ with the detection limit of 0.01 ng mL^−1^. This fabrication procedure is more beneficial than that of the metal electrode as it is prepared from low-cost ITO flat substrate and may be reproduced in batches. This empirical, cost-effective, and pliable electrochemical immunosensor enables a more straightforward and more economic immunoassay for CEA, which can serve the purpose of CEA detection in clinical diagnosis in the future. This strategy could also be used to develop immunosensors for other targets.

## Conflicts of interest

All the authors declares no conflicts of interest.

## Supplementary Material

## References

[cit1] GUIDE TO CANCER Guide to cancer early diagnosis

[cit2] Facts C., Cancer Facts & Figures 2019, 2019

[cit3] Timor-Tritsch I. E., Foley C. E., Brandon C., Yoon E., Ciaffarrano J., Monteagudo A. (2019). *et al.*, New sonographic marker of borderline ovarian tumor: microcystic pattern of papillae and solid components. Ultrasound Obstet. Gynecol..

[cit4] Paner G. P. , ♂♀Cystic and Solid Tumors of the Urachus vs. Gynecologic Tract Tumors: Similarities and Differences, Gynecologic and Urologic Pathology Gynecologic and Urologic Pathology, 2019, pp. 316–330

[cit5] Therapeutic development, 2018, vol. 29(suppl. 10):2018

[cit6] Jain A., Jain A., Gulbake A., Hurkat P., Jain S. K. (2011). Solid tumors: A review. Int. J. Pharm. Pharm. Sci..

[cit7] Wang Y., Pandey M., Ballo M. T. (2019). Integration of Tumor-Treating Fields into the Multidisciplinary Management of Patients with Solid Malignancies. Oncologist.

[cit8] Parihar R., Rivas C., Huynh M., Omer B., Lapteva N. (2019). NK cells expressing a chimeric activating receptor eliminate MDSCs and rescue impaired CAR-T cell activity against solid tumors. Cancer. Immunol. Res..

[cit9] Maeda H., Khatami M. (2018). Analyses of repeated failures in cancer therapy for solid tumors: poor tumor - selective drug delivery , low therapeutic efficacy and unsustainable costs. Clin. Transl. Med..

[cit10] Gavhane Y. N., Shete A. S., Bhagat A. K., Shinde V. R., Bhong K. K., Khairnar G. A. (2011). Solid Tumors : Facts, Challenges and Solutions. Int. J. Pharma Sci. Res..

[cit11] Parihar R., Rivas C., Huynh M., Omer B., Lapteva N., Metelitsa L. S. (2019). *et al.*, NK cells expressing a chimeric activating receptor eliminate MDSCs and rescue impaired CAR-T cell activity against solid tumors. Cancer Immunol. Res..

[cit12] Guedan S., Alemany R. (2018). CAR-T Cells and Oncolytic Viruses : Joining Forces to Overcome the Solid Tumor Challenge. Front. Immunol..

[cit13] Vreeland T. J. , Herbert G. S. and Peoples G. E., Cancer Vaccines for Solid Tumors, 2018

[cit14] Koeppel F., Bobard A., Lefebvre C., Pedrero M., Deloger M., Boursin Y. (2018). Added Value of Whole-Exome and Transcriptome Sequencing for Clinical Molecular Screenings of Advanced Cancer Patients With Solid Tumors. Cancer J..

[cit15] Truta L. A. A. N. A., Sales M. G. F. (2019). Chemical Carcinoembryonic antigen imprinting by electropolymerization on a common conductive glass support and its determination in serum samples. Sens. Actuators, B.

[cit16] Sakamoto S., Putalun W., Vimolmangkang S., Phoolcharoen W. (2017). Enzyme - linked immunosorbent assay for the quantitative/qualitative analysis of plant secondary metabolites. J. Nat. Med..

[cit17] El-Deiry W. S., Goldberg R. M., Lenz H., Shields A. F., Gibney G. T., Tan A. R. (2019). *et al.*, The current state of molecular testing in the treatment of patients with solid tumors. Ca-Cancer J. Clin..

[cit18] Tsekenis G., Chatzipetrou M., Massaouti M., Zergioti I. (2019). Comparative Assessment of Affinity-Based Techniques for Oriented Antibody Immobilization towards Immunosensor Performance Optimization. J. Sens..

[cit19] Pollap A. and Kochana J., Electrochemical Immunosensors for Antibiotic Detection, 2019 10.3390/bios9020061 PMC6628091 31052356

[cit20] Manuscript A., Anal. Methods, 019

[cit21] Fan Y., Shi S., Ma J., Guo Y. (2019). A paper-based electrochemical immunosensor with reduced graphene oxide/thionine/gold nanoparticles nanocomposites modification for the detection of cancer antigen 125. Biosens. Bioelectron..

[cit22] Liu H., Wu X., Zhang X., Burda C., Zhu J. (2012). Gold Nanoclusters as Signal Amplification Labels for Optical Immunosensors. J. Phys. Chem. C.

[cit23] Zhu L., Xu L., Jia N., Huang B., Tan L., Yang S. (2013). *et al.*, Electrochemical immunoassay for carcinoembryonic antigen using gold nanoparticle-graphene composite modified glassy carbon electrode. Talanta.

[cit24] Chen W., Peng J., Ye J., Dai W., Li G., He Y. (2020). Aberrant AFP expression characterizes a subset of hepatocellular carcinoma with distinct gene expression patterns and inferior prognosis. J. Cancer.

[cit25] Kim M.-H., Choi M.-K. (2018). Relationship Between Serum Tumor-related Markers and Dietary Intakes in Korean Healthy Adults. Clin. Nutr. Res..

[cit26] Wang SX. As featured in: 2013;(207890)

[cit27] Basu A., Seth S., Chauhan A. K., Bansal N., Arora K. (2016). Comparative study of tumor markers in patients with colorectal carcinoma before and after chemotherapy. Ann. Transl. Med..

[cit28] Yu S., Li A., Liu Q., Yuan X., Xu H., Jiao D. (2017). *et al.*, Recent advances of bispecific antibodies in solid tumors. J. Hematol. Oncol..

[cit29] Basu A., Seth S., Chauhan A. K., Bansal N., Arora K., Mahaur A. (2016). Comparative study of tumor markers in patients with colorectal carcinoma before and after chemotherapy. Ann. Transl. Med..

[cit30] Dolscheid-Pommerich R. C., Manekeller S., Walgenbach-Brünagel G., Kalff J. C., Hartmann G., Wagner B. S., Holdenrieder S. (2017). Clinical Performance of CEA, CA19-9 CA15-3, CA125 and AFP in Gastrointestinal Cancer Using LOCI™-based Assays. Anticancer Res..

[cit31] Song Z., Yuan R., Chai Y., Yin B., Fu P., Wang J. (2010). Electrochimica Acta Multilayer structured amperometric immunosensor based on gold nanoparticles and Prussian blue nanoparticles/nanocomposite functionalized interface. Langmuir.

[cit32] Bulut I., Arbak P., Coskun A., Balbay O., Annakkaya A. N., Yavuz O., Gülcan E. (2009). Comparison of Serum CA 19.9, CA 125 and CEA Levels with Severity of Chronic. Med. Princ. Prac..

[cit33] Rapicetta C., Lococo F., Carbonelli C., Sverzellati N., Liberata M., Paolo D. (2015). *et al.*, A 79-Year-Old Man With Interstitial Lung Disease and Cryptic Area of High 18 Fluorodeoxyglucose Uptake in Left Upper Lobe. Chest.

[cit34] Hong H. (2020). Clinical characteristics of novel coronavirus disease 2019 (COVID-19) in newborns, infants and children. Pediatr Neonatol..

[cit35] Lippi G., Henry B. M. (2020). Chronic obstructive pulmonary disease is associated with severe coronavirus disease 2019 (COVID-19). Respir. Med..

[cit36] Russell R. (2020). Covid-19 and COPD: A Personal Reflection. Int. J. Chronic Obstruct, Pulm. Dis..

[cit37] Puri N., Sharma V., Tanwar V. K., Singh N., Biradar A. M. (2013). Enzyme-modified indium tin oxide microelectrode array-based electrochemical uric acid biosensor. Prog. Biomater..

[cit38] Aydın EB, Aydın M, Sezgintürk MK. Author ’ s Accepted Manuscript. 2017

[cit39] Liu C., Chen Q., Jiao J., Li S., Hu J., Li Q. (2011). Surface & Coatings Technology Surface modi fi cation of indium tin oxide films with Au ions implantation: Characterization and application in bioelectrochemistry. Surf. Coat. Technol..

[cit40] Links D. A. (2012). Reagentless
amperometric immunosensor for a -1-fetoprotein based on. Anal. Methods.

[cit41] Trindade E. K. G., Silva B. V. M., Dutra R. F. (2019). A probeless and label-free electrochemical immunosensor for cystatin C detection based on ferrocene functionalized-graphene platform.. Biosens. Bioelectron..

[cit42] Biomarker C., Choudhary M., Kumar V., Singh A., Singh M. P., Kaur S. (2013). *et al.*, Graphene Oxide based Label Free Ultrasensitive Immunosensor for Lung. Biosens. Bioelectron..

[cit43] Cho I., Lee J., Kim J., Kang M., Paik J. K., Ku S. (2018). *et al.*, Current Technologies of Electrochemical Immunosensors: Perspective on Signal Amplification. Sensors.

[cit44] Lara S. and Perez-potti A., Applications of Nanomaterials for Immunosensing, 2018 10.3390/bios8040104 PMC6316038 30388865

[cit45] Crespilho F. N., Iost R. M., Travain S. A., Oliveira O. N. (2009). Enzyme immobilization on Ag nanoparticles/polyaniline nanocomposites. Biosens. Bioelectron..

[cit46] Lokina S., Stephen A., Kaviyarasan V., Arulvasu C., Narayanan V. (2014). Cytotoxicity and antimicrobial activities of green synthesized silver nanoparticles. Eur. J. Med. Chem..

[cit47] Chen G. Z., Yin Z. Z., Lou J. F. (2014). Electrochemical immunoassay of escherichia coli O157:H7 using Ag@SiO 2 nanoparticles as labels. J. Anal. Methods Chem..

[cit48] Rocks L., Faulds K., Graham D. (2011). Rationally designed SERS active silica coated silver nanoparticles. Chem. Commun..

[cit49] Thorat N. D., Bauer J., Tofail S. A. M., Gascón Pérez V., Bohara R. A., Yadav H. M. (2020). Silica nano supra-assembly for the targeted delivery of therapeutic cargo to overcome chemoresistance in cancer. Colloids Surf., B.

[cit50] Sharma V., Mishra S. K., Biradar A. M. (2012). Synthesis and electrochemical characterization of myoglobin-antibody protein immobilized self-assembled gold nanoparticles on ITO-glass plate. Mater. Chem. Phys..

[cit51] Bohara R. A., Thorat N. D., Yadav H. M., Pawar S. H. (2014). One-step synthesis of uniform and biocompatible amine functionalized cobalt ferrite nanoparticles: A potential carrier for biomedical applications. New J. Chem..

[cit52] Dong P. V., Ha C. H., Binh L. T., Kasbohm J. (2012). Chemical synthesis and antibacterial activity of novel-shaped silver nanoparticles. Int. Nano Lett..

[cit53] Branda F. , The Sol-Gel Route to Nanocomposites, 2007

[cit54] Tuteja S. K., Kukkar M., Kumar P., Paul A. K., Deep A. (2014). Synthesis and Characterization of Silica-Coated Silver Nanoprobe for Paraoxon pesticide Detection. Bionanoscience.

[cit55] Ranjgar A., Norouzi R., Zolanvari A., Sadeghi H. (2013). Characterization and Optical Absorption Properties of Plasmonic Nanostructured Thin Films. Arm. J. Phys..

[cit56] Wang W., Zhao J., Short M., Zeng H. (2015). Real-time *in vivo* cancer diagnosis using Raman spectroscopy. J. Biophotonics.

[cit57] Yang J., Rudi J., Gunasekaran S. (2012). Nanoscale Indium tin oxide-coated glass modified with reduced graphene oxide sheets and gold nanoparticles as disposable working electrodes for dopamine sensing in meat samples. Nanoscale.

[cit58] Balahura L., Staden R. S., Frederick J., Staden V., Aboul-enein H. Y. (2018). Advances in immunosensors for clinical applications. J. Immunoassay Immunochem..

[cit59] Huang K. J., Niu D. J., Xie W. Z., Wang W. (2010). A disposable electrochemical immunosensor for carcinoembryonic antigen based on nano-Au/multi-walled carbon nanotubes-chitosans nanocomposite film modified glassy carbon electrode. Anal. Chim. Acta.

[cit60] Kemmegne-mbouguen J. C., Ngameni E., Baker P. G., Tesfaye T., Kgarebe B., Iwuoha E. I. (2014). Carcinoembryonic Antigen Immunosensor Developed with Organoclay Nanogold Composite Film. Int. J. Electrochem. Sci..

[cit61] Wang H., Wang Y., Zhang Y., Wang Q., Ren X., Wu D. (2016). *et al.*, Photoelectrochemical Immunosensor for Detection of Carcinoembryonic Antigen Based on 2D TiO 2 Nanosheets and Carboxylated Graphitic Carbon Nitride. Nat. Publ. Gr..

[cit62] Zhong Z., Wu W., Wang D., Wang D., Shan J., Qing Y. (2010). *et al.*, Biosensors and Bioelectronics Nanogold-enwrapped graphene nanocomposites as trace labels for sensitivity enhancement of electrochemical immunosensors in clinical immunoassays: Carcinoembryonic antigen as a model. Biosens. Bioelectron..

[cit63] He X., Yuan R., Chai Y., Shi Y. (2008). A sensitive amperometric immunosensor for carcinoembryonic antigen detection with porous nanogold film and nano-Au/chitosan composite as immobilization matrix. J. Biochem. Biophys. Methods.

[cit64] Sun X., Ma Z. (2013). Electrochemical immunosensor based on nanoporpus gold loading thionine for carcinoembryonic antigen. Anal. Chim. Acta.

[cit65] Shi W., Ma Z. (2011). A novel label-free amperometric immunosensor for carcinoembryonic antigen based on redox membrane. Biosens. Bioelectron..

[cit66] Lv P., Min L., Yuan R., Chai Y., Chen S. (2010). A novel immunosensor for carcinoembryonic antigen based on poly (diallyldimethylammonium chloride) protected prussian blue nanoparticles and double-layer nanometer-sized gold particles. Microchim. Acta.

[cit67] Yuan Y., Yuan R., Chai Y., Zhuo Y., Mao L., Yuan S. (2010). A novel label-free electrochemical immunosensor for carcinoembryonic antigen detection based on the [Ag–Ag_2_O]/SiO_2_ nanocomposite material as a redox probe. J. Electroanal. Chem..

[cit68] Gao X., Zhang Y., Wu Q., Chen H., Chen Z., Lin X. (2011). One step electrochemically deposited nanocomposite film of chitosan-carbon nanotubes-gold nanoparticles for carcinoembryonic antigen immunosensor application. Talanta.

[cit69] Wang Y., Wang Y., Liu B., Deng Y., Xu D., Pang D. (2017). Target-triggered signal turn-on detection of prostate specific antigen based on metal- enhanced fluorescence of Ag@SiO_2_@SiO_2_ - RuBpy composite nanoparticles. Nanotechnology.

[cit70] Fatema K. N., Liu Y., Cho K. Y., Oh W. C. (2020). Comparative study of electrochemical biosensors based on highly efficient mesoporous ZrO_2_-Ag-G-SiO_2_and In_2_O_3_-G-SiO_2_ for rapid recognition of E. coli O157:H7. ACS Omega.

[cit71] Fathima H., Paul L., Thirunavukkuarasu S., Thomas K. G. (2020). Mesoporous Silica-Capped Silver Nanoparticles for Sieving and Surface-Enhanced Raman Scattering-Based Sensing. ACS Appl. Nano Mater..

[cit72] Yang Y., Zhu J., Zhao J., Weng G. J., Li J. J., Zhao J. W. (2019). Growth of Spherical Gold Satellites on the Surface of Au@Ag@SiO_2_ Core-Shell Nanostructures Used for an Ultrasensitive SERS Immunoassay of Alpha-Fetoprotein. ACS Appl. Mater. Interfaces.

[cit73] Yang Y., Huang H., Wang X., Zhang L., Hao A., Shi Z. (2020). *et al.*, Silica-coated silver nanoparticles decorated with fluorescent CdTe quantum dots and DNA aptamers for detection of tetracycline. ACS Appl. Nano Mater..

[cit74] Krishnan S., Chinnasamy T., Veerappan S. (2014). Dual labeled Ag@SiO_2_ core–shell nanoparticle based optical immunosensor for sensitive detection of E. coli. Mater. Sci. Eng., C.

[cit75] Gan T., Li J., Xu L., Guo S., Zhao A., Sun J. (2020). Multishell Au@Ag@SiO_2_ nanorods embedded into a molecularly imprinted polymer as electrochemical sensing platform for quantification of theobromine. Microchim. Acta.

[cit76] Zhang Y., Sun H., Gao R., Zhang F., Zhu A., Chen L. (2018). *et al.*, Facile SERS-active chip (PS@Ag/SiO_2_/Ag) for the determination of HCC biomarker. Sens. Actuators, B.

